# Altered Effective Connectivity Within a Thalamocortical Corollary Discharge Network in Individuals With Schizophrenia

**DOI:** 10.1093/schbul/sbae232

**Published:** 2025-01-24

**Authors:** Matthew Lehet, Beier Yao, Ivy F Tso, Vaibhav A Diwadkar, Jessica Fattal, Jacqueline Bao, Katharine N Thakkar

**Affiliations:** Psychology, Michigan State University, East Lansing, MI, 48824, United States; Psychology, Chatham University, Pittsburgh, PA, 15232, United States; Psychology, Michigan State University, East Lansing, MI, 48824, United States; Schizophrenia and Bipolar Disorder Program, McLean Hospital, Belmont, MA, 02478, United States; Psychiatry, Harvard Medical School, Boston, MA, 02115, United States; Psychiatry and Behavioral Health, The Ohio State University, Columbus, OH, 43210, United States; Psychiatry and Behavioral Neuroscience, Wayne State University, Detroit, MI, 48202, United States; Psychology, Northwestern University, Evanston, IL, 60208, United States; Psychology & Neuroscience, Duke University, Durham, NC, 27708, United States; Psychology, Michigan State University, East Lansing, MI, 48824, United States; Psychiatry and Behavioral Medicine, Michigan State University College of Human Medicine, Grand Rapids, MI, 49503, United States

**Keywords:** psychosis, dynamic causal modeling, DCM, fMRI, visual remapping, double-step

## Abstract

**Background and Hypothesis:**

Sequential saccade planning requires corollary discharge (CD) signals that provide information about the planned landing location of an eye movement. These CD signals may be altered among individuals with schizophrenia (SZ), providing a potential mechanism to explain passivity and anomalous self-experiences broadly. In healthy controls (HC), a key oculomotor CD network transmits CD signals from the thalamus to the frontal eye fields (FEF) and the intraparietal sulcus (IPS) and also remaps signals from FEF to IPS.

**Study Design:**

Here, we modeled fMRI data using dynamic causal modeling (DCM) to examine patient-control differences in effective connectivity evoked by a double-step (DS) task (30 SZ, 29 HC). The interrogated network was formed from a combination of (1) functionally identified FEF and IPS regions that robustly responded on DS trials and (2) anatomically identified thalamic regions involved in CD transmission. We also examined the relationship between clinical symptoms and effective connectivity parameters associated with task modulation of network pathways.

**Study Results:**

Network connectivity was indeed modulated by the DS task, which involves CD transmission. More importantly, we found reduced effective connectivity from thalamus to IPS in SZ, which was further correlated with passivity symptom severity.

**Conclusions:**

These results reaffirm the importance of IPS and thalamocortical connections in oculomotor CD signaling and provide mechanistic insights into CD alterations and consequently agency disturbances in schizophrenia.

## Introduction

Disturbances in a sense of self, and specifically in agency, have long been considered at the root of many symptoms of schizophrenia.^1^ However, not understanding the mechanisms underlying an aberrant sense of self poses a barrier to treatment development. Corollary discharge (CD) signals—copies of motor signals sent to sensory areas in the brain—permit predictions about the sensory consequences of action; thus, CD provides important sensorimotor information that supports a sense of agency (ie, “I am in control of my actions.”). Sensory input that matches CD-based predictions is tagged as having been self-generated, whereas mismatches are flagged as having been externally generated. Consequently, disruptions in CD signaling may contribute to an altered sense of self and agency in schizophrenia.^2^

**Figure 1.**
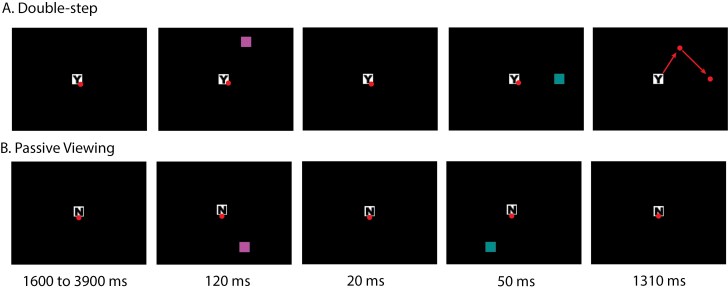
Double-step task. The circular dots indicate gaze location, and the arrows represent saccades. These do not appear on the task screen. The stimuli presented on DS and PV trials were identical, but the two trial types differed in task instruction regarding movement execution. A letter presented at the center of the initial fixation square (Y or N) provided trial instructions. (A) On double-step trials, the letter in the central square was Y and participants were instructed to look at the targets in the order they appeared as quickly and accurately as possible. The fixation was on screen for 1.6-3.9 s before two targets were presented sequentially. The targets appeared equiprobably in one of 6 locations for 120 ms. Then after 20 ms, a second target appeared in one of the adjacent locations for 50 ms. Targets always formed an equilateral triangle with the fixation. (B) Passive viewing trials were identical except an N in the central fixation indicated participants should not look at the targets. The timeline and stimuli presentation were the same as double-step trials. The two targets are in different colors to help indicate the sequence of their appearance. The order of colors of the targets was counterbalanced across participants. The design also included fixation trials consisted of an N in the central square that stayed in the center of the screen for 9.4-13.6 s. No targets appeared on these trials.

There is robust evidence for altered CD in schizophrenia from several sensorimotor modalities,^3–5^ including the oculomotor system, and studying CD related to saccades has unique advantages over other sensorimotor systems. First, well-established and rigorous psychophysics paradigms can measure the influence of CD on visual perception and eye movements.^6^ Corollary discharge supports critical oculomotor functions, including the planning of sequential saccades.^7^ Because CD signals contain the kinematics of the imminent saccade (eg, direction, amplitude), sequential saccades can be planned in parallel: preparation of a second saccade can incorporate information about the anticipated gaze location following the first saccade.^8^ The effect of CD on sequential saccade planning can be measured using the double-step (DS) task,^7^ where participants are asked to look at two rapidly flashed sequential targets in the dark (Figure 1A). Because there is no sensory information available after the first saccade, participants must rely on the extra-retinal signals provided by CD to make an accurate second saccade. In prior studies, we have shown altered DS task performance behaviorally in schizophrenia that is consistent with a reduced influence of CD.^9,10^

A second advantage of investigating oculomotor CD is that neurophysiology studies in nonhuman primates have used the DS task to articulate a neural circuit involved in the generation and transmission of oculomotor CD signals, including saccade neurons in the superior colliculus (SC), visual neurons in the frontal eye fields (FEF), and mediodorsal thalamus (MD).^11^ Visual neurons have a receptive field—an area of the sensory space in which stimuli evoke a neuronal response. Visual neurons in several cortical regions, including FEF and the intraparietal sulcus (IPS), predictively shift their receptive fields during saccade planning to the future position of gaze, ie, remapping.^12^ CD in the SC-MD-FEF pathway contributes to remapping, which may be proximal to accurate DS performance. Remapping signals may also transmit from FEF to IPS directly.^12,13^ Human lesion studies have shown that damage to thalamus^14,15^ and IPS^16^ lead to deficits in the DS task. In primates, temporary inactivation of MD disrupts DS task performance in a manner consistent with a loss of CD, and similar to the performance observed in schizophrenia patients.^9,10^

In this study, we used dynamic causal modeling (DCM) of functional MRI (fMRI) timeseries data acquired while participants performed the DS task. Unlike task-based fMRI analysis that only examines activation differences with no directional information, DCM has the unique advantage of gaining *mechanistic* understanding of the dynamic *causal* influences that multiple brain regions have on each other.^17^ Here, we aimed to understand whether and, if so, how key pathways that transmit oculomotor CD signals are altered in schizophrenia. Regions of interest included FEF, IPS, and subdivisions of thalamus that transmit oculomotor CD signals.^12,18,19^ Our goals were also guided by the literature on functional and structural thalamocortical disconnections in schizophrenia,^20–28^ and our previous finding of reduced structural connectivity of an MD-FEF pathway in schizophrenia that was associated with impaired trans-saccadic perception (which relies on CD) and psychotic symptoms.^29^ Based on these studies, we hypothesized reduced effective connectivity in this oculomotor CD network in people with schizophrenia relative to healthy participants—particularly from thalamus to cortical oculomotor regions, and an association between network connectivity and clinical symptom severity.

## Methods

### Participants

Thirty individuals with schizophrenia (SZ) and thirty healthy controls (HC) were recruited through community advertisements, existing research registries and subject pools, and outpatient mental health facilities. An electronic version of the Structured Clinical Interview for DSM-5^30^ was used for diagnostic assessment. Participants with a history of neurological disorders, DSM-5 moderate or severe substance use disorder within the previous six months, history of head injury with loss of consciousness longer than one hour, or vision that was not normal or corrected-to-normal were excluded. Healthy controls with a family history of schizophrenia spectrum disorders or bipolar disorder, or a personal history of DSM-5 Axis-I disorders or psychotropic use were also excluded. One HC was excluded due to poor task performance (see Supplementary Methods). Chlorpromazine equivalent doses^31–33^ were calculated for SZ. Demographic and clinical data for all included participants are reported in Table 1. Groups were matched on age, race, sex assigned at birth, and handedness. Participants provided written informed consent and were reimbursed for their participation. The Michigan State University Institutional Review Board approved the study.

**Table 1.** Demographic Information

	HC (*N* = 29)	SZ (*N* = 30)		
	Mean (SD)	Mean (SD)	Statistic	*P*-value
Age (years)	37.38 (11.64)	39.60 (10.69)	*t*(57) = 0.76	.448
Race	21 White, 3 African American, 2 Asian/Indian, 1 Multiracial, 2 Other	20 White, 7 African American, 2 Multiracial, 1 Native American/Alaska Native	*χ* ^2^ = 0.04	.844
Sex (female/male)	12 F/17 M	9 F/21 M	*χ* ^2^ = 0.41	.522
WTAR	111.15 (5.98)	105.41 (10.64)	*t*(53) = 2.43	.019
Education (years)	16.76 (2.68)	14.25 (1.74)	*t*(57) = 4.28	<.001
Handedness	52.93 (60.51)	48.16 (60.24)	*t*(57) = 0.30	.763
Duration of Illness (years)		17.87 (10.29)		
CPZ equivalent (mg)		373.16 (354.00)		
SANS		21.67 (15.23)		
SAPS		20.43 (21.61)		
IPASE	72.87 (17.38)	132.31 (41.48)	*t*(48) = 6.51	<.001
SAPP		2.79 (2.75)		

The chi-square test for Race collapsed racial categories into white/other categories (HC: 21/8; SZ: 20/10) in order to meet the frequency requirement of the test.

Abbreviations: CPZ, chlorpromazine; HC, healthy controls; IPASE, Inventory of Psychotic-like Anomalous Self-Experiences lifetime score; SANS, Scale for the Assessment of Negative Symptoms total score; SAPS, Scale for the Assessment of Positive Symptoms total score; SAPP, Scale for the Assessment of Passivity Phenomena lifetime score; SZ, individuals with schizophrenia or schizoaffective disorder; WTAR, Wechsler Test for Adult Reading. Handedness was measured with the Edinburgh handedness inventory (−100 = complete left-handedness, 100 = complete right-handedness).

### Assessments

Clinical symptoms were assessed using the Scale for the Assessment of Positive Symptoms (SAPS)^34^ and the Scale for the Assessment of Negative Symptoms total score (SANS).^35^ The Inventory of Psychotic-like Anomalous Self-Experiences lifetime score (IPASE)^36^ and Scale for the Assessment of Passivity Phenomena lifetime score (SAPP)^37^ were used to measure agency-related experiences. Premorbid IQ was assessed using the Wechsler Test of Adult Reading,^38^ and handedness was assessed using the Edinburgh Handedness Scale.^39^ See Supplementary Methods for details of missing assessments.

### DS Task

#### Experimental Paradigm

We used a block design fMRI activation paradigm. The DS task (Figure 1) comprised two equiprobable trial types: DS and passive viewing (PV) trials, which were interleaved with fixation (ie, rest) periods. The stimuli presented on DS and PV trials were identical, but the two trial types differed in task instruction regarding movement execution. Both trials began with the presentation of a central fixation square (0.5° × 0.5° visual angle) of variable duration (between 1.6 and 3.9 s), followed by two square targets (T1 and T2; 0.5° × 0.5° visual angle) which were flashed one after the other at a distance of 10° from the fixation square. T1 was presented equiprobably at one of six possible locations; the location of T2 was constrained such that T1 and T2 were presented at adjacent positions that formed an equilateral triangle with the fixation square. T1 was flashed for 120 ms. Onset of T2 was 20 ms after T1 offset and was flashed for 50 ms. The target squares were cyan and magenta, with the mapping of these colors onto T1 and T2 counterbalanced across participants. A letter presented at the center of the initial fixation square (Y or N) provided trial instructions. On DS trials (indicated with a “Y” in the central fixation square), participants were instructed to look at the targets in the order they appeared as quickly and accurately as possible. On PV trials (indicated with an “N” in the central fixation square), participants were instructed to maintain fixation throughout the trial (ie, not look towards the flashing targets). The PV condition served as a control condition: it involved identical visual input to the DS condition, but without saccade commands or related CD. On fixation trials (also indicated with an “N” in the central fixation square), no stimuli were presented, and participants were instructed to maintain central fixation.

Participants typically completed 5 runs of the DS task, although four participants had fewer runs (see Supplementary Methods for details on this missing data). Each run comprised 7 blocks each of DS trials, PV trials, and fixation. Double-step and PV blocks had a duration of 11.1-19.6 s; fixation blocks had a duration of 9.4-13.6 s. Double-step and PV blocks consisted of 4 trials. Catch trials were included to assess attention (see Supplementary Methods). On three blocks in each run (one fixation, one DS, and one PV block), the fixation square was enlarged to 3° × 3° visual angle (by a magnitude of 6) during one trial in the block. When this occurred, participants were instructed to make a key press with their index finger.

#### Apparatus and Setup

Participants viewed a screen in the scanner bore by way of a mirror attached to the head coil (viewing distance 60 cm from screen to eye), with the task stimuli projected onto it by a Hyperion digital projection system (Psychology Software Tools, Inc., Pittsburgh, PA; refresh rate 60 hz, resolution 1024 × 768, with a 30.55° × 22.92° visual angle). The right eye was tracked by an Eyelink 1000 plus eye tracker (SR Research, Ottawa, Ontario, Canada). We used Psychophysics^40^ and EyeLink^41^ toolboxes via MATLAB (MathWorks, Portola Valley, CA) to present stimuli and collected key press responses via a BrainLogics fiber optic 5-button response glove (Psychology Software Tools, Inc., Pittsburgh, PA).

#### Data Analysis

On each trial, a drift correction was applied to the eye position data using mean eye position within a 100 ms window centered around T1 onset. Due to poor eye-tracking data quality, trial accuracy was evaluated in our main analyses by determining whether a task-relevant saccade was present or not. Saccades were identified using the automated EyeLink procedure (velocity threshold: 30°/s, acceleration threshold: 8,000°/s,^2^ motion threshold: 0.1°). Saccades were considered task-relevant if they had a minimum amplitude of 2° visual angle, a minimum reaction time of 80 ms after T1 onset, and a maximum duration of 250 ms. Double-step trials with one or more task-relevant saccades and PV trials without task-relevant saccades were considered accurate. Trials with 20% or more missing eye position data after T1 onset were excluded from analysis. To examine group differences in task performance, accuracy was entered into a mixed 2 × 2 ANOVA, with condition (DS vs PV) as the within-subject variable and group (SZ vs HC) as the between-subject variable. See Supplementary Methods for evaluation of more precise saccade kinematics.

### fMRI Data Collection and Analysis

#### Data Acquisition

Scans were acquired on a 3.0 T Signa HDx MR scanner (GE Healthcare, Waukesha, WI) using an eight-channel receive only GE head coil. Whole-brain T2*-weighted echo planar images with blood-oxygen level-dependent (BOLD) contrast (150 volumes; 38 slices per volume; interleaved acquisition; TR 2.2 s; TE 25 ms; field of view 220 × 220 × 114 mm; flip angle 78°; 64 × 64 × 38 matrix; voxel size 3.4375 × 3.4375 × 3.0) oriented in a transverse plane were acquired. The first four volumes were discarded to allow for T1 equilibration effects. A whole-brain three-dimensional fast-field echo T1-weighted scan (256 slices; TI 40 ms; TE 3.8 ms; flip angle 8°; field of view, 180 × 256 × 256 mm; voxel size: 1 × 1 × 1 mm) was acquired for within-subject registration.

#### Preprocessing

Functional data were preprocessed and analyzed using a combination of SPM 12 (http://www.fil.ion.ucl.ac.uk/spm/software/) and AFNI (https://afni.nimh.nih.gov/). In SPM, the functional acquisitions were spatially realigned to correct for head motion using rigid body transformations, and a mean functional image was generated. Next, slice time correction was applied to temporally align the signal in each slice to the middle slice. The anatomical image was co-registered to the mean functional image using normalized mutual information and then segmented and normalized to Montreal Neurological Institute space using unified segmentation as implemented in SPM 12.^42^ These normalization parameters were then applied to the functional images. Functional images were spatially smoothed with a Gaussian kernel (full width at half maximum = 6 mm). Finally, volumes were despiked using AFNI’s 3Ddespike function.

#### First-Level General Linear Models

Statistical analysis followed a two-level procedure within the framework of the general linear model (GLM). First-level statistical analyses modeled blocks of DS trials, blocks of PV trials, and catch trials. Regressors for DS and PV blocks were created by convolving the onsets and durations of each block with a canonical hemodynamic response function. Double-step and PV blocks containing catch trials were split around the catch trial into shorter blocks that did not include the catch trial. Catch trials and any individual DS or PV trials resulting from splitting blocks around catch trials were modeled using the trial onset and trial duration convolved with a canonical hemodynamic response function. Fixation served as an implicit baseline. Nuisance regressors included delta functions modeling key press responses to catch trials convolved with the hemodynamic response, a timeseries from a sphere of cerebrospinal fluid around the posterior commissure (radius of 10 mm) to remove noise in subcortical structures, and three movement regressors capturing movement over time (by quantifying total displacement from the origin across acquisitions in three regions: center, left posterior inferior, left posterior superior) derived from the motion fingerprinting toolbox.^43,44^ Temporal autocorrelation in the fMRI data was modeled using autoregressive modeling of the first order by prewhitening the data. Data were also high-pass filtered during prewhitening with a cutoff cycle length of 128 s.

We isolated activation related to executing saccades to sequential targets by examining the regions where activity was greater during DS blocks than PV blocks. Given that the PV condition may involve inhibition of a prepotent response to look at the peripheral targets, we also examined activation that was greater for PV than DS trials.

#### Second-Level GLMs

Whole-brain second-level random-effects analysis was conducted on first-level contrasts using one-way, paired, and independent sample *t-*tests. These second-level contrast images were computed for HC, SZ, the combined sample, and comparing HC vs SZ. To examine how clinical assessments related to the DS greater than PV contrast, we ran separate analyses with each clinical measure (SAPS, SANS, SAPP, IPASE for SZ and IPASE for HC) as a covariate in the independent sample *t*-tests. Finally, to examine how groups differed on the DS greater than PV contrast, we used an *F* contrast to identify the group differences in the contrast of DS > PV. Second-level analyses were corrected for multiple comparisons using an uncorrected *P* < .001 and a cluster-wise threshold of *P* < .05.

**Figure 2.**
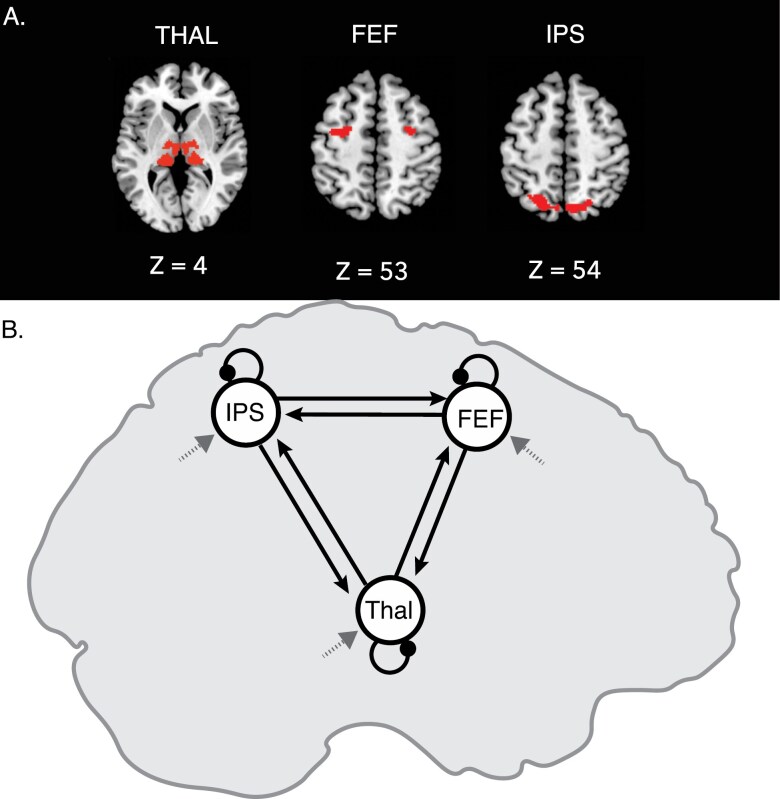
Regions of interest and DCM model design. (A) We examined activation within and causal interactions between three bilateral regions of interest implicated in the transmission and utilization of CD signals in the oculomotor control system. Subregions of the thalamus (THAL) that relay CD signals based on previous literature (pulvinar, mediodorsal, ventrolateral anterior, and ventrolateral posterior nuclei) were identified using masks from a previously published atlas. Frontal eye fields (FEF) and intraparietal sulcus (IPS) were defined using the DS > PV contrast across groups (thresholded at uncorrected *P* < 1*10^−13^ for FEF and *P* < 1*10^-15^ for IPS) and prior knowledge of the anatomical locations of these regions including labels from the automated anatomical labeling atlas in MRIcron. Bilateral ROIs were combined into a single mask in each region (ie, treated as a single ROI). (B) In the DCM model, driving activation was modeled at all regions (dashed arrows). All connections were modeled as between-regions as well as all self-inhibition connections as within-regions. Task activity (PV, DS, and catch trials) was modeled as a delta function for each event. Modulation was modeled on all connections (including between-region connections and self-connections within regions) for blocks of DS trials.

To further examine group differences in activation in DS and PV conditions within regions known to be involved in saccade control and planning, we used an ROI analysis approach in three brain regions (FEF, IPS, and thalamic subregions; see Figure 2A). Cortical ROIs were guided by prior knowledge of the anatomical locations of these regions including labels from the automated anatomical labeling atlas in MRIcron^45^ and functionally defined based on activation in the combined sample from the DS > PV contrast, thresholded using uncorrected *P*-values (FEF: *P* < 1 × 10^−13^ and IPS: *P* < 1 × 10^−15^). Uncorrected threshold was higher in IPS to separate this cluster from adjacent clusters of activation. Thalamic subregions (pulvinar, mediodorsal, ventrolateral anterior, and ventrolateral posterior nuclei; THAL) were defined based on previously published probability maps^46^ and combined into a single ROI. Bilateral activation within each ROI was combined across hemispheres during signal extraction. Local percentage signal change was extracted from each of these three ROIs for DS and PV trials. For each ROI, repeated-measures ANOVAs were conducted to investigate the effects of condition (DS vs PV), diagnostic group, and their interaction.

#### DCM Analysis

To assess effective connectivity between our ROIs during the DS task, we used the DCM framework (DCM for fMRI using SPM12).^47–49^ We examined how the engagement of oculomotor systems during DS trials modulated directed or effective connectivity within and between regions and investigated how this modulation may differ between groups.

DCM permits identification of causal influence between regions of interest by building generative models of predicted neural activity^48,50^ and then optimizing across effective connectivity parameters within these models. These effective connectivity parameters quantify how regional changes in activity affect rates of activity change within and across regions in the network. Additionally, they quantify the modulation of those connections based on task characteristics (eg, generating sequential saccades). First, a forward generative model of the BOLD response is created by inferring neural activity based on the driving input from the task, influence from connected regions, self-connections, and modulation by task condition. Then, the predicted BOLD signal is compared to the fMRI time series. By iteratively optimizing parameters in the generative model and regenerating the predicted BOLD signal, an optimal set of effective connectivity parameters can be identified. This optimization (inversion) balances the fit (between the predicted response and the fMRI time series) and the complexity (the change between each prior parameter value and the posterior estimated parameter value) of the model. The optimized fit of these parameters characterizes the dynamic causal influences of our neural network.

Here, we use this computational theoretical framework to examine similarities and differences between HC and SZ in effective connectivity within an oculomotor network (including FEF, THAL, and IPS) thought to utilize CD in service of motor planning during sequential saccades. The responses in each ROI were summarized with the primary eigenvector, having adjusted the data for confounds (ie, the null space of the effects of interest). We set the slice timing model in our DCM to half the TR, in line with published recommendations.^48^ All connections between and within regions were modeled (solid arrows in Figure 2B). A new GLM was created for the purpose of defining onsets to the DCM model with a regressor for task—all trials in the experiment (DS trials, PV trials, catch trials) were modeled as delta functions convolved with a hemodynamic response function. The task regressor modeled every trial (including the visual input and consequent sensorimotor integration) and was used as driving input to all three regions (dashed arrows in Figure 2B; see Supplementary Results in Supplementary Table S3 for an analysis suggesting that driving input to all regions is likely given the data). A second regressor for DS blocks was defined to differentiate whether each trial involved eye movements or not, and thus examined specific modulation related to rapid consecutive eye movement planning and execution. Connections associated with the task (instantiated in an “A matrix”) should be interpreted as the average effective connectivity (during all task trials) whereas modulation parameters (instantiated in a “B matrix”) are specific to DS trials (where saccades were initiated) and add or subtract from that average.

In order to optimize our search space of models for each participant, we inverted a full model for each participant and used a second-level Parametric Empirical Bayes (PEB) analysis (optimized over the A and B matrices), followed by Bayesian Model Comparison (using the function spm_dcm_peb_bmc), to estimate average task-related and DS modulation parameter values across both groups and to identify how SZ differed from HC in their average and modulation parameters.^47–49^ We focused our reporting on parameters with greater than 95% posterior probability (labeled as “credible” from here onwards). For connections between regions, the parameter units are in hertz. In other words, an effective connectivity of 0.2 means that the influence from one region to the other would take about 5 seconds to manifest (a relatively strong connection in the context of fMRI). Credible positive parameter values (“excitatory” connections) reflect effective connectivity where increased activity in the source region leads to an increased rate of change in activation in the receiving region. Credible negative parameter values (“inhibitory” connections) reflect effective connectivity where increased activity in the source region results in decreased rates of changes in activation in the receiving region. Self-connections reflect within-region inhibition where the initial (default) parameter value starts at −0.5 (log scale) and positive parameters reflect increase in inhibition whereas negative connections reflect decrease in inhibition (relative to the starting value). Modulation parameters reflect additive changes in effective connectivity on DS blocks relative to the mean effective connectivity throughout the task.

We further probed the importance of connections from thalamus to cortex during the DS task by comparing four families of models: (1) models that had modulation on DS blocks from both THAL to FEF and THAL to IPS, (2) models that had modulation on DS blocks from THAL to FEF, (3) models that had modulation on DS blocks from THAL to IPS, and (4) all other models. The likelihood that each family of models fit the data was assessed with the spm_dcm_peb_bmc_fam command for each group separately.

Finally, we investigated relationships between oculomotor CD network connectivity and symptom scores for SZ (SAPS, SANS, SAPP, and IPASE) and HC (IPASE) as well as CPZ equivalent dosages. Five separate PEB analyses were run with each symptom score as a regressor in the PEB as well as another with CPZ as a regressor. Given our interest in how modulation on DS blocks was related to symptoms that might index agency disturbances, these PEB analyses were optimized over the B modulation matrix. This resulted in a measure of modulation due to DS and how that modulation was affected by symptom scores for each of these PEB analyses. Again, we reported credible parameters for these analyses. The parameters reflecting the contribution of symptom scores (and CPZ) are in units of hertz per unit change in score. By convention, these scores are normalized to have zero mean and unit variance.

## Results

### Behavioral Results

Analysis of usable eye-tracking data showed that participants overall adhered to the task instructions (see Supplementary Results for details of missing eye position data). On DS trials, participants made task-related saccades on the majority of trials (HC mean = 91.30%, SE = 2.63%; SZ mean = 72.36%, SE = 5.49%). Conversely, on PV trials, participants did not make task-related saccades on the majority of trials (HC mean = 87.50%, SE = 2.73%; SZ mean = 80.56%, SE = 4.36%). We found a main effect of group (*F*(1,57) = 16.86, *P* < .001, *η*^2^ = 0.08) on task accuracy such that HC were more accurate across instruction conditions. We found no main effect of condition (*F*(1,57) = 0.22, *P* = .644, *η*^2^ < 0.01), and no group × condition interaction (*F*(1,57) = 1.60, *P* = .210, *η*^2^ = 0.02). These results suggest good adherence to task instructions in both groups, albeit with better performance in HC (see Supplementary Results for an analysis of spatial accuracy on DS trials). Overall, participants were quite accurate at detecting and responding to catch trials (M = 96.2% SE = 0.86; M_HC_ = 97.93% SE = 0.88; M_SZ_ = 95.8% SE = 1.45). Importantly, catch trial accuracy was high in both HC and SZ and did not differ between groups *t*(57) = 1.26, *P* = .213.

### fMRI GLM Results

Whole-brain analyses are reported in Supplementary Results. For the ROI analysis, percent signal change for each participant in each ROI for the DS and PV conditions (see Supplementary Figure S3) were entered into a 2 factor (group: HC vs SZ) × 2 factor (condition: DS vs PV) mixed ANOVA. We found main effects of condition in all three regions (FEF: *F*(1,57) = 186.26, *P* < .001, *η*^2^ = 0.09; THAL: *F*(1,57) = 30.89, *P* < .001, *η*^2^ = 0.02; IPS: *F*(1,57) = 208.47, *P* < .001, *η*^2^ = 0.09). There were no main effects of group (FEF: *F*(1,57) = 0.14, *P* = .712, *η*^2^ <= 0.010; THAL: *F*(1,57) = 0.14, *P* = .708, *η*^2^ <= 0.010; IPS: *F*(1,57) = 0.18, *P* = .675, *η*^2^ <= 0.010), nor were there any interactions between group and condition (FEF: *F*(1,57) = 0.05, *P* = .819, *η*^2^ <= 0.010; THAL: *F*(1,57) = 0.91, *P* = .345, *η*^2^ <= 0.010; IPS: *F*(1,57) = 0.09, *P* = .770, *η*^2^ <= 0.010). We did not observe any correlations between clinical measures and differences between DS and PV activation in any ROIs. (Supplementary Table S9).

### DCM Results

**Figure 3.**
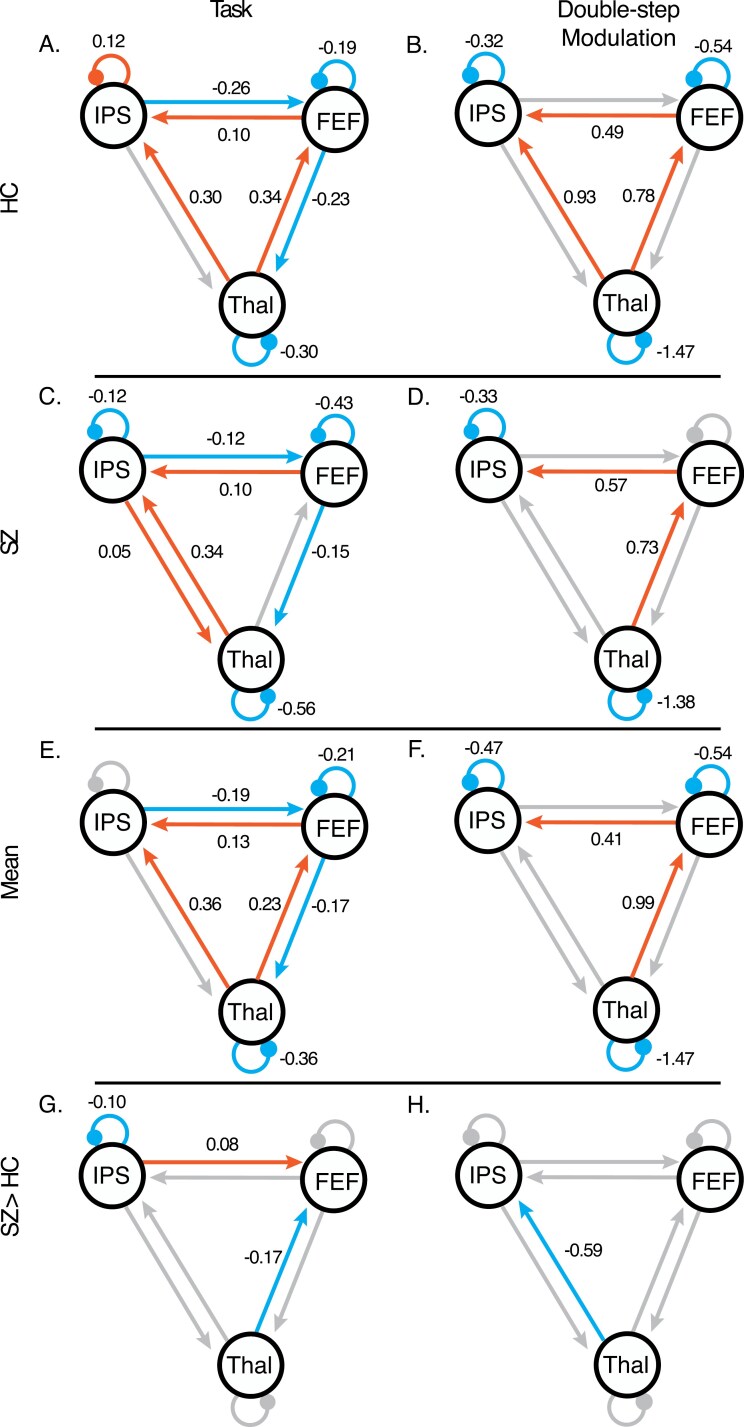
DCM results of the PEB analysis for each group, including HC (top row), SZ (second row), across groups (third row), and group differences (fourth row). Parameter values are indicated above each connection that is 95% likely to be nonzero based on free energy. Between-region connections with positive parameter values indicate excitatory effective connectivity. Between-region connections with negative parameter values indicate inhibitory effective connectivity parameters that are 95% likely. Self-connections with negative parameter values indicate less self-inhibition of a region than the parameter default whereas positive parameter values indicate more self-inhibition in a region. The left column presents the task-related activity (including DS, PV, and catch trials) and the right column presents the modulation on blocks of DS trials. We therefore show (A) task-related activity in HC; (B) modulation on DS blocks for HC; (C) task-related activity in SZ; (D) modulation on DS blocks for SZ; (E) mean group effective connectivity during all task trials across groups; (F) mean modulation across groups associated with blocks of DS trials; (G) the mean group differences (SZ > HC) across all conditions; (H) group differences in modulation associated with blocks of DS trials. Abbreviations: FEF, frontal eye field; IPS, intraparietal sulcus; THA, thalamic nuclei associated with CD.

Figure 3 shows the results of the DCM analysis from HC (top row), SZ (second row), and the group analysis that includes the mean across groups (third row) and the group differences (bottom row). Each analysis shows mean effective connectivity during all task trials (PV and DS trials; left column) and modulation by instructions to execute sequential saccades (DS blocks; right column). Parameter values and probabilities can be found in Supplementary Results, Supplementary Tables S4, S5, and S6.

The mean across groups (Figure 3 third row) identifies robust coordination within the specified oculomotor CD network throughout the task, in line with predictions. During all task trials, across both groups we found self-inhibition in the FEF and THAL was reduced relative to the default priors used in DCM for fMRI (Figure 3E; Supplementary Table S6 “Mean Effective Connectivity”). We found credible excitatory influences from the THAL to FEF and IPS, as well as from FEF to IPS. These findings align with a functional model of the network whereby CD signals (putatively from the SC) are relayed from the thalamus to FEF and then remapping signals from FEF are sent to other cortical areas (including IPS). Finally, we found inhibition from IPS to FEF and from FEF to THAL. On DS blocks, we found less self-inhibition in all regions and increased excitatory connections from THAL to FEF and from FEF to IPS (Figure 3F; Supplementary Table S6 “Mean Modulation”).

Group differences in effective connectivity (Figure 3 fourth row) included task-related connectivity where the SZ group showed less self-inhibition in the IPS, less excitation from THAL to FEF, and less inhibition from IPS to FEF (Figure 3G; Supplementary Table S6 “Group Differences in Mean Connectivity”). On DS blocks, SZ showed less excitation than HC from THAL to IPS (Figure 3H; Supplementary Table S6 “Group Differences in Modulation”). These group differences are evident when comparing the credible connections between the HC (Figure 3 top row, Supplementary Table S4) and the SZ (Figure 3 second row, Supplementary Table S5) groups. Combined, these findings indicate reduced excitatory connectivity from the thalamus to cortex and reduced modulation of thalamocortical connections by sequential saccade execution.

In line with the group differences PEB analysis, the winning model family for the HC group (with 100.00% probability) was the family of models that included modulation of effective connectivity from THAL to IPS and from THAL to FEF. However, for the SZ group, the winning family of models (with 82.38% probability) was those with connections between THAL to FEF but not THAL to IPS. The family of models with both THAL to IPS and THAL to FEF was second most likely (with 17.61 % probability). Other families were improbable (with 0.00% probabilities).

**Figure 4.**
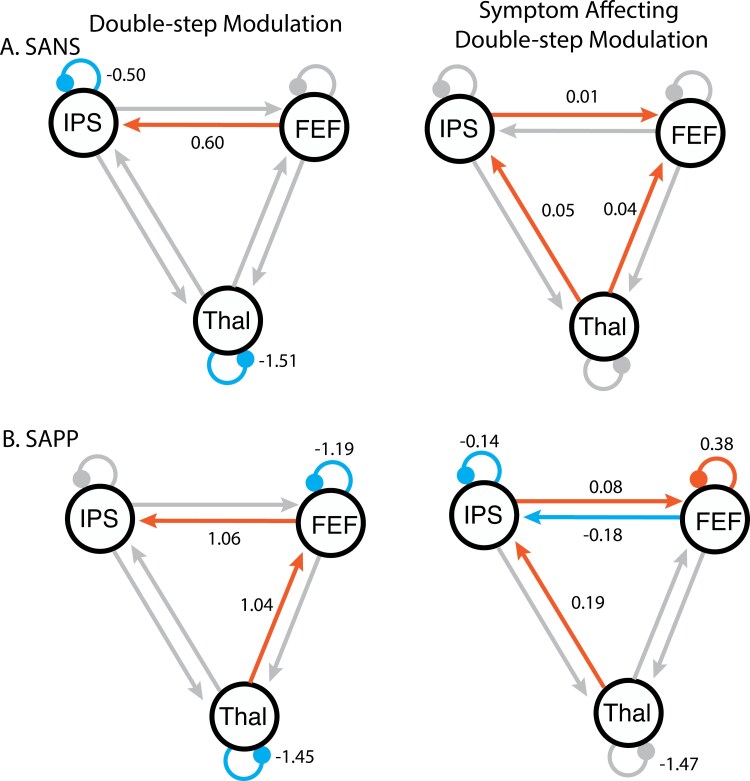
Symptom analyses within SZ. The PEB was optimized across the modulation of DS trials so the left column represents mean modulation on DS blocks due to sequential saccade planning and execution. The right column represents how this modulation was affected by variation in symptom scores across participants. Symptom scores were run in separate models and included: (A) the SANS; (B) the Scale for the Assessment of Passivity Phenomena lifetime score (SAPP). Scale for the Assessment of Positive Symptoms (SAPS), and Inventory of Psychotic-like Anomalous Self-Experiences lifetime score (IPASE) in both SZ and HC were also run but did not show credible relationships with DS modulation. Abbreviations: FEF, frontal eye field; IPS, intraparietal sulcus; THAL, thalamic nuclei associated with CD.

We also examined the influence of medication, symptom severity, and anomalous self-experiences on the modulation of effective connectivity by executing sequential saccades (Figure 4). Specifically, the analysis shows the modulation due to DS (Figure 4, left column) and—most importantly—the influence of symptom scores on that modulation (Figure 4, right column). Parameter values and probabilities for all symptom scores (SANS, SAPS, SAPP, IPASE in SZ, and IPASE in HC) as well as CPZ equivalent scores in SZ can be found in Supplementary Results Supplementary Tables S7 and S8. There was no effect of CPZ on DS modulation. In this section, we highlight associations between symptoms and modulation on connections from THAL to both FEF and IPS and from FEF to IPS, given their putative roles in relaying oculomotor CD signals. Full symptom analyses are reported in Supplementary Results. SANS and SAPP scores are credibly associated with DS modulation. Higher SANS scores were related to increased excitatory modulation from THAL to FEF and from THAL to IPS. Higher SAPP scores were related to increased excitatory modulation from THAL to IPS and reduced excitatory modulation from FEF to IPS.

These results can be interpreted in the context of the group differences DCM analysis, whereby HC showed more DS modulation of the excitatory THAL to IPS connection and more task-related excitatory connectivity from THAL to FEF. Somewhat counterintuitively, SZ with higher SANS and SAPP scores showed DS modulation of effective thalamocortical connectivity that was more similar to HC. On the other hand, SZ showed reduced DS modulation of the excitatory FEF to IPS connection compared to HC, and this was particularly prominent in those with more severe passivity symptoms.

## Discussion

In this study, we examined effective connectivity of a key thalamocortical network that is critically involved in relaying oculomotor CD signals in people with schizophrenia and matched controls. DCM allowed us to corroborate findings from nonhuman primate and human lesion studies, and we found that a pathway from thalamus to frontal and parietal eye movement regions is indeed modulated by the task condition that requires CD signaling for accurate performance (DS saccades). Our results contribute to this basic science literature by providing new insights into thalamo-parietal involvement in CD signaling and specific cortical pathways supporting visual remapping. Importantly, we observed reduced effective connectivity in the pathway from thalamus to an oculomotor region in the parietal, but not frontal, cortex in people with schizophrenia. Because CD signals are considered a basic sensorimotor mechanism of agency, our results suggest that altered functioning in *specific* thalamocortical pathways is associated with, and may contribute to, complex self-related symptoms of schizophrenia.

Effective connectivity analyses in this study shed light on network functioning within a putative saccade control and CD network in people with and without schizophrenia. Collapsed across groups, mean connectivity during all task trials (Figure 3E) largely recapitulates involvement of thalamus, IPS, and FEF in visual attention and saccade control.^51–53^ Importantly, the degree to which the instruction to execute a saccade (which should involve CD transmission) modulates effective connectivity in controls (Figure 3B) is consistent with data from neurophysiology and lesion studies of CD transmission associated with saccadic eye movements and visual remapping. Specifically, we found credible excitatory modulation of the thalamus to FEF and FEF to IPS connections. MD is critical for transmitting CD signals from motor neurons in the SC to visual neurons in the FEF; when these MD neurons are inactivated, monkeys execute sequential saccades in a way that would be predicted by a failure of CD signals to accurately inform future gaze position.^11^ FEF contains many neurons that encode both current and future visual receptive fields (ie, shifting RFs) immediately before a saccade,^8^ and CD signals routed via MD support remapping of visual FEF neurons.^54^ FEF has been posited as the main source of remapped visual signals to other cortical visual regions, including the IPS^12^; however, this finding has not been directly established. Indeed, IPS has reciprocal connections to FEF,^13^ and other lines of work indicate that IPS can also send remapping signals to the FEF.^55–57^ That we found credible modulation of the FEF to IPS pathway (but not IPS to FEF) highlights the importance of communication from FEF to IPS in executing sequential saccades; a function critically reliant on effective CD signals.

Another pathway thought to transmit oculomotor CD signals goes from SC to pulvinar (in the thalamus) to IPS and extrastriate visual cortex^12,58^; however, to our knowledge, this pathway has only been studied in the context of smooth pursuit eye movements in nonhuman primates.^59,60^ Nevertheless, given the overlap in smooth pursuit and saccade systems,^61^ the SC-THAL-IPS pathway may also transmit CD signals necessary for saccades. Indeed, thalamic lesions in humans reduce parietal updating of the visual processing in response to executing the first saccade in the DS task,^15^ suggesting that thalamus sends CD signals related to the first saccade in the DS paradigm to IPS either directly or indirectly. Similarly, parietal lesions in humans led to significant impairments in completing the second saccade in the DS task.^16,62^ Data from the current study support the importance of thalamo-parietal pathways in transmitting the CD signals necessary for sequential saccades.

The group differences in the effective connectivity pattern converge to show the importance of IPS and thalamocortical circuits. During all task trials, there was less excitation from thalamus to FEF, less inhibitory connection from IPS to FEF, and less self-inhibition in IPS in SZ (Figure 3G). This is consistent with our previous finding of impaired structural connectivity in the MD-FEF pathway in SZ,^29^ as well as the larger functional and structural thalamocortical dysconnectivity literature.^20–28^ During the DS trials, we found less excitatory modulation of the thalamus to IPS connection in SZ relative to HC (Figure 3H). In other words, IPS activity accelerated more slowly in response to signals from thalamus. Findings from the family model comparison analysis corroborated findings from the PEB analysis, supporting this thalamocortical dysconnection in SZ: while the winning model for controls included DS modulation of pathways from thalamus to both IPS and FEF, the winning model for people with schizophrenia only included the thalamus to FEF pathway.

Reduced modulation of this thalamus-IPS connection is consistent with findings of reduced volume in thalamic subregions with connections to the parietal cortex^63^ and reduced functional connectivity between thalamus and inferior parietal lobule (IPL)^64,65^ in SZ. Although previous work has highlighted the role of THAL-FEF connectivity in CD signaling associated with saccadic eye movements,^29^ there are almost certainly multiple pathways transmitting CD signals related to an eye movement.^11^ Current findings highlight the importance of THAL-IPS pathway in CD signaling generally and its role in putative alterations in CD signaling in SZ and, in turn, the pathway’s possible association with self-related symptoms of SZ.^66,67^ IPS receives CD signals related to eye, head, and arm movement commands via thalamus.^58^ Therefore, the THAL-IPS pathway is well positioned as a key neural underpinning of our sense of agency. Indeed, right IPL disruption using TMS lead to increased agency misattribution in reaching movements in nonclinical participants.^68^ Moreover, reduced volume^69^ and increased activation of IPL^37,70^ in SZ have been associated with passivity symptoms and positive symptoms^71^ more generally.

The notion that altered modulation of this network is related to passivity symptoms comes from current findings that SAPP scores are related to task modulation on both FEF to IPS and thalamus to IPS connections, but in different ways. SAPP scores were related to reduced excitatory modulation from FEF to IPS. In other words, IPS activity accelerated more slowly in those with more severe passivity symptoms in response to putative remapping signals from FEF. This is consistent with the notion that FEF conveys CD-informed signals to IPS, and alterations in this communication are associated with passivity symptoms.^66,67^ More importantly, this finding suggests that the relationship between CD alterations and agency disturbances is not restricted to communication between lower motor centers and the cortex, but could occur at cortico-cortical connections that further propagate CD-informed computations, such as the FEF-IPS pathway. On the other hand, SZ with more severe passivity symptoms showed increased excitatory connection from THAL to IPS. In other words, IPS activity accelerated more quickly in response to putative CD signals from THAL in the more symptomatic SZ, compared with SZ with less severe passivity symptoms. In sum, there seems to be a nuanced relationship between passivity symptoms and alterations at different stages of CD-informed oculomotor computation within the THAL-FEF-IPS network. In considering these relationships, we must note, however, that the current outpatient sample has a limited range of passivity symptoms, and current findings may not reflect the full relationship between symptom severity and network connectivity. Another point worth clarifying is that although DCM is able to model causal influences between brain regions and the causal effect of specific task conditions on neural activities, the symptom effect analysis is different due to its correlational nature—the association with symptom severity is driven by individual differences rather than experimental manipulation. Therefore, a causal connection between effective connectivity and symptom level can only be established through longitudinal designs and/or direct brain stimulation.

While these findings can be interpreted in the context of the physiological hypothesis positing a role for altered CD in schizophrenia risk or symptom presentation, they can also be interpreted in a broader explanatory computational and biologically plausible theory of psychosis: the predictive coding account. This theory postulates that psychosis may be a result of imbalanced integration of prior beliefs and sensory signals.^72^ More specifically, aberrant modulation of the precision of prediction errors (ie, mismatches between predictions and sensory data) has been hypothesized to drive faulty inferences about the external world, especially in the context of active vision and perceptual inference.^73,74^ Mathematically, precision refers to the variability of predictions (supported by CD) and ensuing prediction errors. Physiologically, precision is thought to be encoded by excitation-inhibition balance or postsynaptic gain of neuronal populations encoding prediction errors (eg, superficial pyramidal cells).^75^ In our DCM model, modulation of effective connectivity on DS trials can be interpreted as reflecting the modulatory effect on precision of prediction errors. Under this framework, the group difference in thalamus-IPS effective connectivity supports a failed modulation of precision of saccade-related prediction errors.

The current study has several limitations. First, the quality of the eye-tracking data did not allow us to measure the degree to which second saccade accuracy was informed by extra-retinal information provided by CD, which we have reported and replicated in previous studies.^9,10^ These analyses require precise measures of saccade kinematics that could not be ascertained from this data. Consequently, we were not able to relate trial-by-trial measures of CD to activation patterns and network connectivity patterns. Secondly, the current patient sample has a rather limited range of agency disturbances, and even fewer were actively experiencing passivity symptoms during the study. Therefore, future research utilizing a sample with more prominent and active passivity symptoms is needed to test the relationship between less effective CD transmission and agency disturbances more directly. Third, most of the patients in this study were taking antipsychotics, making it difficult to rule out medication effects. Nevertheless, the absence of a significant effect of CPZ equivalent dosage on the network effective connectivity argues against our results being driven by medication status. Finally, while DCM can provide insights into directed (ie, causal) connections in the brain, there are model assumptions and limitations regarding how the analysis accurately represents various complexities of physiology that bear on the reliability and meaning of these interpretations—challenges that are endemic to all models of biology and physiology.^76^ These limitations aside, we believe that current findings generated important insights into the underlying mechanisms of CD alterations in SZ and point to potential directions for future work. For example, future studies could look into the effect of interventions targeting specific CD pathways in patients experiencing agency disturbances in particular.

In conclusion, we found that relative to HC, SZ had less effective connectivity within a putative oculomotor CD network, particularly in the thalamo-parietal pathway. Current findings also extend translational findings in the network involved in transmitting CD signals by adding in vivo human data to the literature. It is worth pointing out that the traditional GLM analysis was not able to identify group differences in neural activity. In other words, connectivity, but not activation differences, distinguished patients from controls. These findings highlight the advantage of DCM and suggest that effective connectivity analysis may provide more salient information about altered brain function in schizophrenia and how these effects relate to our understanding of some of the most pathognomonic but understudied symptoms in the illness.

## Supplementary Material

sbae232_suppl_Supplementary_Tables_S3-S9_Figures_S3
