# Supplementary material for: Altered Effective Connectivity Within a Thalamocortical Corollary Discharge Network in Individuals With Schizophrenia
Source: Schizophr Bull. 2025 Jan 24;51(6):1637–50. doi: 10.1093/schbul/sbae232 (PMC12597492; doi:10.1093/schbul/sbae232)
Supplement: sbae232_suppl_Supplementary_Tables_S3-S9_Figures_S3 [file sbae232_suppl_supplementary_tables_s3-s9_figures_s3.docx]

**Supplementary Methods**

**Missing data**

Clinical assessment data was missing from a number of participants. IPASE was not administered to five healthy controls (HC) and four participants with schizophrenia (SZ). Two other participants (one HC and one SZ) were only missing a single item on IPASE, so within-person mean imputation was used to calculate total scores and they were included in the analyses. SAPS and SANS were both missing from a single SZ. SAPP lifetime scores were missing from two SZ. There are no published CPZ equivalent doses for some newer antipsychotic medications; thus CPZ equivalent doses were not able to be calculated for four SZ participants. One SZ and three HC did not have WTAR collected. Four participants completed fewer runs of the MRI task (1 HC had 4 runs, 1 HC and 1 SZ had 3 runs, 1 SZ had 2 runs).

**Exclusion based on task performance**

To identify participants who were not attending to the task we included catch trials in the experimental design (see Methods section for details). Participants who performed worse than a cutoff of three standard deviations below the overall mean (72%) were excluded. Only one participant (HC) did not achieve this level of accuracy and they were excluded from all further analyses.

**Behavioral analysis of eye tracking data**

Our main analysis focused on the presence or absence of task-relevant saccades. Additional analyses of saccade kinematics were limited by the quality of data. For many participants poor calibration (e,g. due to MRI-compatible corrective eye glasses and limited flexibility in positioning the head relative to camera) or pupil trace loss led to missing or spatially inaccurate eye data. We excluded trials if 20% or more eye position samples after T1 onset were missing.

To examine more precise metrics of task performance, we analyzed task-relevant saccades to identify saccades to T1 and T2. Saccades to T1 were identified by the comparison of three angles (see **Figure S1**), all originating at the saccade start point. Angle one comprised two arms connecting the origin with saccade endpoint and T1. Angle two comprised two arms connecting the origin with saccade endpoint and T2. Angle three comprised two arms connecting the origin with T1 and T2. The algorithm for classifying a saccade as directed to T1 or T2 (or neither) are summarized in Figure S1. The saccade was classified as towards T1 if one of two criteria were met: (1) if both angle one and two were smaller than angle three and angle two was greater than angle one (i.e., the saccade trajectory was in between T1 and T2, but closer to T1 than to T2); (2) if angle two was bigger than angle three and angle one was smaller than half of angle three (i.e., the saccade was to the side of T1 away from T2, but within a small enough angle - half of angle three - to be considered towards T1). Similarly, the saccade was classified as towards T2 given one of two scenarios: (1) if both angle one and two were smaller than angle three and angle two was greater than angle one; (2) if angle one was greater than angle three and angle two was less than half of angle three.

Saccades that followed the initial saccade to T1 were further classified as either corrective saccades toward T1 (that landed closer to T1 than T2) or saccades to T2 (that landed closer to T2 than T1). Saccades that landed within the fixation window around the fixation were considered returns to fixation.

To identify group and condition dependent differences in missing data, the average rate of excluded trials on double-step and passive viewing trials were submitted to a 2 x 2 ANOVA, with condition (double-step vs passive viewing) as the within-subject variable and group (SZ vs HC) as the between-subject variable. We also assessed behavior on double-step trials with usable eye position data across groups. We identified on each trial whether participants 1) looked at both T1 and T2 in order, 2) looked at T2 first, 3) looked at T1 first but not to T2 4) did not make a saccade, 5) made an initial saccade to a location other than T1 or T2. Within each of these different subclasses of performance we compared group performance with an independent samples t-test. We also used an independent samples t-test to compare the rates of trials where saccades to T1 or T2 were incorrectly made on passive viewing trials.

**Supplementary Results**

**Behavioral results**

Missing eye position data

Trials with 20% or more missing data of eye position after T1 onset were excluded from further analysis (N_total_ = 5203 total trials across groups and trial types). The number of excluded trials differed substantially among participants (min=0% of trials excluded, max = 97.78% of trials excluded). In the ANOVA, we found no main effect of group F(1,57) = 0.12, p = 0.729, η^2^ < 0.01. We did find significant differences between conditions F(1,57) = 71.04, p < 0.001, η^2^ = 0.18; rates of excluded trials were higher in the passive viewing condition (M = 46.59%, SE = 3.88, N_total_ = 3583 trials) than double-step condition (M = 21.52%, SE = 3.19, N_total_ = 1620 trials). This was likely the result of participants closing their eyes more on passive viewing trials. We also found a group x condition interaction F(1,57) = 7.91, p = 0.007, η^2^ = 0.02. This interaction resulted from more missing data on passive viewing trials among HC (M = 49.73%,SE = 5.36, N_total_ = 1884 trials) than among SZ (M = 43.56%, SE = 5.64, N_total_ = 1699 trials), but HC had less missing data on double-step trials (M = 16.10%, SE = 3.95, N_total_ = 600 trials) than did SZ (M = 26.75%, SE = 4.85, N_total_ = 1020 trials).

Spatial accuracy on double-step trials

On double-step trials with usable data (i.e., trials missing < 20% eye position data, N_total_ = 6129 trials), participants most frequently (average of 30.90% of usable trials, N_total_ = 2226 trials) looked at both T1 and T2 in order. The most frequent error they made was looking at T2 first (average of 22.66% of usable trials, N_total_ = 1400 trials). Other types of errors included looking at T1 first but not following up with a saccade to T2 (average of 21.18% of usable trials, N_total_ = 1045 trials), not making a saccade (average of 18.33% of usable trials, N_total_ = 806 trials), or making an initial saccade to a location other than T1 or T2 (average of 6.92% of usable trials, N_total_ = 378). Double-step performance across groups and statistical comparisons are shown in **Table S1**. HC were more likely to make saccades to T1 and then T2 (p<0.001) whereas SZ were more likely to look at T1 but not follow up with a saccade to T2 (p<0.001), and SZ were more likely to not make a saccade at all (p=0.003). The groups did not differ in the percent of trials where they made saccades to T2 first (p=0.399), or trials where they made an incorrect saccade (p=0.195).

Participants did not make a saccade on most passive viewing trials with usable eye data (N_total_ = 4166 trials), but we further examined trials where they did make a saccade by mistake to identify any potential group differences. The percentage of usable passive viewing trials that had neither saccade to T1 nor T2 did not significantly differ (t(57)=1.162, p=0.250) between HC (M = 88.61%, SE = 2.64, N_total_ = 1773 trials) and SZ (M = 83.14%, SE = 3.86, N_total_ = 1981 trials), suggesting that the rate of trials where saccades to T1 or T2 were incorrectly made were infrequent and equivalent between groups.

**GLM analysis: Whole brain**

Double-step > passive viewing contrast

We identified widespread activation in both groups for the contrast of double-step greater than passive viewing (see **Figure S2A** and **S2B**). In both groups, visual regions showed robust activity as did regions involved in vision and saccade planning such as frontal eye fields, inferior and superior parietal, and supplementary eye fields. We additionally found activation in inferior frontal and temporal-parietal junction regions that might be associated with directed attentional control. The significant regions identified were activated robustly enough that they formed a singular cluster and as such only the first 10 most activated peaks are reported in Supplementary Results **Table S2**.

Passive viewing > double-step

We found different regions activated in the two groups for the contrast of passive viewing greater than double-step. For HC (see **Figure S2C**), we found a region of right occipital cortex that was more responsive to passive viewing than double-step blocks. For SZ (see **Figure S2D**) we found an area of left occipital cortex bordering inferior parietal as well as bilateral activation in superior temporal regions.

Group differences

There were no significant group differences in either the double-step > passive viewing or passive viewing > double-step contrasts.

Symptom correlations

We examined how clinical assessments were related to task activity by including each clinical assessment as a covariate in the contrast of double-step greater than passive viewing. In each of these analyses for SZ, the clinical assessments did not significantly relate to activity in any regions: even at a very liberal threshold of p<0.05 uncorrected, no clusters were significant at a clusterwise threshold of p < 0.05. In HC, IPASE scores showed a significant relationship to double-step greater than passive viewing contrast in superior medial frontal regions that survived a threshold of p<0.001 uncorrected with a clusterwise threshold of p < 0.05 (see **Figure S2E**).

**Initial PEB analysis of C matrix to determine driving input**

To determine which regions should have driving input modeled in our final DCM analysis, we first optimized a PEB analysis over the C matrix with a single regressor of all 1s across both groups. This provided the mean activation across both groups for driving input to all three regions (see **Table S3**). All three regions showed mean values in the PEB that significantly differed from zero and therefore driving input to all three regions were included in the analysis.

**DCM symptom analysis**

We saw increased excitatory modulation from IPS to FEF associated with higher SANS scores and higher SAPP scores. We also saw changes in within-region self-inhibitory modulation such that higher SAPP scores were associated with less within region self-inhibitory modulation within IPS but increased self-inhibitory modulation in FEF. While these results may not reflect altered corollary discharge they do suggest symptom relationships with effective connectivity modulation on blocks of double-step trials.


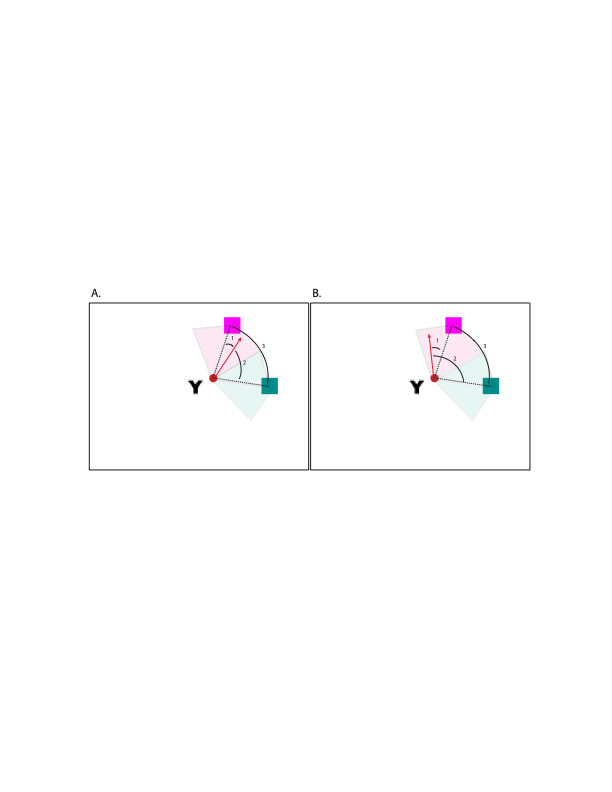


**Figure S1.** Schematic illustrating how task relevant saccades were classified as directed to T1 (magenta square) or T2 (teal square). The red arrow represents the saccade and was not displayed to participants. Likewise, the shaded regions indicate areas where saccade endpoints would be classified as directed towards T1 (magenta shading), or T2 (teal shading), or neither (outside of the shaded space). From the saccade start point (red dot), three angles were calculated. Angle one comprised two arms connecting the origin with saccade endpoint and T1. Angle two comprised two arms connecting the origin with saccade endpoint and T2. Angle three comprised two arms connecting the origin with T1 and T2. Panels A & B illustrate the two cases where the saccade is classified as towards T1: A) the case where both angle one and two were smaller than angle three and angle two was greater than the angle one (i.e., the saccade was in between T1 and T2, and closer to T1 than it is to T2); B) the case where angle two was bigger than angle three and angle one was smaller than half of angle three (i.e., the saccade was to the side of T1 away from T2, but within a small enough angle - half of angle three - to be considered towards T1).

**Figure S2.** Significant GLM whole-brain analyses results. A) HC contrast showing double-step > passive viewing blocks. B) SZ contrast showing double-step > passive viewing blocks. C) HC contrast showing passive viewing > double-step blocks. D) SZ contrast showing passive viewing > double-step blocks. E) HC IPASE scores as a covariate in the contrast of double-step > passive viewing blocks. Slices show MNI coordinate Z = -20 to 60 in increments of 10. Only significant regions are shown.

**Figure S3.** Region of interest analyses including A) frontal eye field (FEF), B) intraparietal sulcus (IPS), and C) thalamic nuclei associated with corollary discharge (THAL). The percent signal change in the two conditions of interest (passive viewing and double step blocks) is shown using box plots and violin plots to characterize the distribution of values. Significant differences between conditions were found in all three regions but no group differences or interactions between conditions were observed.

| **Table S1.** Behavioral breakdown of double-step trials | | | | |
| --- | --- | --- | --- | --- |
| Trial Type | HC Mean (SE) | SZ Mean (SE) | t-stat (df=57) | P value |
| Mean N (SE) | 111.52 (5.69) | 96.5 (6.74) | 1.70 | 0.095 |
| Correct saccades to T1 and T2 (%) | 42.50 (4.48) | 19.69 (3.83) | 3.87 | <0.001 |
| Saccade to T2 first (%) | 23.71 (1.54) | 21.64 (1.86) | 0.85 | 0.399 |
| Correct saccade to T1 but no correct saccade to T2 (%) | 19.71 (2.81) | 22.60 (3.22) | 4.05 | <0.001 |
| No saccade (%) | 8.70 (2.62) | 27.64 (5.49) | 3.08 | 0.003 |
| Incorrect first saccade (to neither T1 nor T2) (%) | 5.39 (1.16) | 8.42 (1.98) | 1.31 | 0.195 |
|  |  |  |  |  |

| **Table S2.** GLM results | | | | | | | |
| --- | --- | --- | --- | --- | --- | --- | --- |
|  | Cluster Size | Cluster wise | Peak | Peak MNI Coordinates (mm) | | |  |
| Analysis | (Voxels) | P (FWE-corr) | T-stat | x | y | z | Region |
| HC: Double-step > Passive Viewing | | | | | | | |
|  | 55290 | <0.001 | 15.5 | 6 | -76 | 2 | R. Lingual |
|  |  |  | 15.08 | 8 | -66 | -8 | R. Lingual |
|  |  |  | 13.17 | -6 | -72 | -6 | L. Lingual |
|  |  |  | 12.41 | 8 | -70 | -26 | R. Cerebellum |
|  |  |  | 11.87 | -22 | -68 | 52 | L. Superior Parietal |
|  |  |  | 11.6 | 0 | -72 | 12 | L. Calcarine |
|  |  |  | 11.58 | -20 | -74 | -14 | L. Cerebellum |
|  |  |  | 11.38 | -8 | -72 | -20 | L. Cerebellum |
|  |  |  | 10.94 | -22 | -54 | -12 | L. Lingual |
|  |  |  | 10.86 | 18 | -76 | -18 | R. Cerebellum |
| SZ: Double-step > Passive Viewing | | | | | | | |
|  | 46194 | <.001 | 12.05 | -6 | -72 | -14 | L. Cerebellum |
|  |  |  | 11.82 | 4 | -74 | -12 | Vermis |
|  |  |  | 11.43 | -12 | -64 | -8 | L. Lingual |
|  |  |  | 11.26 | 40 | -40 | 42 | R. Inferior Parietal |
|  |  |  | 11.1 | -8 | -68 | -2 | L. Lingual |
|  |  |  | 11.1 | 6 | -66 | 0 | R. Lingual |
|  |  |  | 10.87 | 18 | -70 | 50 | R. Superior Parietal |
|  |  |  | 10.79 | 12 | -74 | 16 | R. Calcarine |
|  |  |  | 10.68 | -22 | -28 | -4 | L. Hippocampus |
|  |  |  | 10.64 | 20 | -64 | -12 | R. Calcarine |
| HC: Passive Viewing > Double-step | | | | | | | |
|  | 157 | 0.044 | 6.96 | 32 | -94 | -14 | R. Inferior Occipital |
| SZ: Passive Viewing > Double-step | | | | | | | |
|  | 230 | 0.014 | 6.55 | -42 | -6 | -16 | L. Mid Temporal |
|  |  |  | 4.63 | -34 | 10 | -26 | L. Superior Temporal Pole |
|  |  |  | 4.3 | -42 | -20 | 0 | L. Superior Temporal |
|  |  |  | 4.03 | -44 | -10 | -6 | L. Superior Temporal |
|  |  |  | 3.82 | -38 | 4 | -22 | L. Superior Temporal Pole |
|  | 212 | 0.02 | 5.96 | -44 | -74 | 36 | L. Mid Occipital |
|  |  |  | 4.56 | -36 | -78 | 42 | L. Inferior Parietal |
|  | 208 | 0.021 | 5.8 | 46 | -2 | -16 | R. Mid Temporal |
|  |  |  | 4.46 | 38 | 6 | -22 | R. Superior Temporal Pole |
|  |  |  | 3.88 | 34 | 12 | -26 | R. Superior Temporal Pole |
| HC>SZ: Double-step > Passive Viewing | | | | | | | |
|  | 1813 | 0.015 | 17.49 | 10 | 42 | 18 | R. Anterior Cingulum |
|  |  |  | 13.75 | -26 | 18 | -12 | L. Insula |
|  |  |  | 12.6 | 8 | 32 | 28 | R. Anterior Cingulum |
|  |  |  | 11.45 | -6 | 26 | 32 | L. Anterior Cingulum |
|  |  |  | 10.92 | -8 | 36 | 28 | L. Anterior Cingulum |
|  |  |  | 9.89 | -10 | 24 | 14 | L. Caudate |
|  |  |  | 9.51 | -28 | 0 | -8 | L. Putamen |
|  |  |  | 9.27 | 8 | 28 | 16 | R. Anterior Cingulum |
|  |  |  | 8.5 | -40 | -6 | -16 | L. Mid Temporal |
|  |  |  | 8.3 | -22 | 10 | -4 | L. Putamen |
| HC: IPASE as a covariate in Double-step > Passive Viewing | | | | | | | |
|  | 145 | 0.048 | 5.43 | 8 | 24 | 10 | R. Anterior Cingulate |
|  |  |  | 4.29 | 14 | 38 | 0 | R. Anterior Cingulate |
|  |  |  | 4.22 | 6 | 42 | 4 | R. Anterior Cingulate |
|  |  |  | 3.84 | 14 | 30 | 2 | R. Anterior Cingulate |

| **Table S3.** Group DCM Analysis across C matrix | | | |
| --- | --- | --- | --- |
| Connection Type | Connection | PEB Probability | PEB parameter |
| Mean Driving Input | to THAL | 0.97 | 0.04 |
| Mean Driving Input | to IPS | 1.00 | 0.42 |
| Mean Driving Input | to FEF | 1.00 | 0.28 |
| Note: The PEB model was optimized over the C matrix, so A and B parameters are not estimated. | | | |

| **Table S4.** HC DCM parameters | |  |  |  |
| --- | --- | --- | --- | --- |
| Connection type | Connection | | BMA Probability | BMA parameter |
| Mean Effective Connectivity | From THAL to THAL | | 1.00 | -0.30 |
| Mean Effective Connectivity | From THAL to IPS | | 1.00 | 0.30 |
| Mean Effective Connectivity | From THAL to FEF | | 1.00 | 0.34 |
| Mean Effective Connectivity | From IPS to THAL | | 0.68 | 0.03 |
| Mean Effective Connectivity | From IPS to IPS | | 0.99 | 0.12 |
| Mean Effective Connectivity | From IPS to FEF | | 1.00 | -0.26 |
| Mean Effective Connectivity | From FEF to THAL | | 1.00 | -0.23 |
| Mean Effective Connectivity | From FEF to IPS | | 0.95 | 0.10 |
| Mean Effective Connectivity | From FEF to FEF | | 1.00 | -0.19 |
| Connection type | Connection | | BMA Probability | BMA parameter |
| Modulation due to Double-step | From THAL to THAL | | 1.00 | -1.47 |
| Modulation due to Double-step | From THAL to IPS | | 1.00 | 0.93 |
| Modulation due to Double-step | From THAL to FEF | | 1.00 | 0.78 |
| Modulation due to Double-step | From IPS to THAL | | 0.00 | -0.00 |
| Modulation due to Double-step | From IPS to IPS | | 0.98 | -0.32 |
| Modulation due to Double-step | From IPS to FEF | | 0.00 | -0.00 |
| Modulation due to Double-step | From FEF to THAL | | 0.72 | 0.15 |
| Modulation due to Double-step | From FEF to IPS | | 1.00 | 0.49 |
| Modulation due to Double-step | From FEF to FEF | | 1.00 | -1.10 |
| Note: The PEB and BMA model was not optimized over the C matrix, so C parameters are not estimated in the BMA. | | | | |

| **Table S5.** SZ DCM parameters | |  |  |  |
| --- | --- | --- | --- | --- |
| Connection type | Connection | | BMA Probability | BMA parameter |
| Mean Effective Connectivity | From THAL to THAL | | 1.00 | -0.56 |
| Mean Effective Connectivity | From THAL to IPS | | 1.00 | 0.34 |
| Mean Effective Connectivity | From THAL to FEF | | 0.84 | 0.07 |
| Mean Effective Connectivity | From IPS to THAL | | 0.97 | 0.05 |
| Mean Effective Connectivity | From IPS to IPS | | 1.00 | -0.12 |
| Mean Effective Connectivity | From IPS to FEF | | 1.00 | -0.12 |
| Mean Effective Connectivity | From FEF to THAL | | 1.00 | -0.15 |
| Mean Effective Connectivity | From FEF to IPS | | 0.98 | 0.10 |
| Mean Effective Connectivity | From FEF to FEF | | 1.00 | -0.43 |
| Connection type | Connection | | BMA Probability | BMA parameter |
| Modulation due to Double-step | From THAL to THAL | | 1.00 | -1.38 |
| Modulation due to Double-step | From THAL to IPS | | 0.00 | -0.00 |
| Modulation due to Double-step | From THAL to FEF | | 1.00 | 0.73 |
| Modulation due to Double-step | From IPS to THAL | | 0.00 | 0.00 |
| Modulation due to Double-step | From IPS to IPS | | 0.95 | -0.33 |
| Modulation due to Double-step | From IPS to FEF | | 0.00 | -0.00 |
| Modulation due to Double-step | From FEF to THAL | | 0.00 | -0.00 |
| Modulation due to Double-step | From FEF to IPS | | 1.00 | 0.56 |
| Modulation due to Double-step | From FEF to FEF | | 0.84 | -0.40 |
| Note: The PEB and BMA model was not optimized over the C matrix, so C parameters are not estimated in the BMA. | | | | |

| **Table S6.** Group DCM analysis. | | | |
| --- | --- | --- | --- |
| Connection Type | Connection | BMA Probability | BMA parameter |
| Mean Effective Connectivity | From THAL to THAL | 1.00 | -0.36 |
| Mean Effective Connectivity | From THAL to IPS | 1.00 | 0.36 |
| Mean Effective Connectivity | From THAL to FEF | 1.00 | 0.23 |
| Mean Effective Connectivity | From IPS to THAL | 0.67 | 0.02 |
| Mean Effective Connectivity | From IPS to IPS | 0.88 | 0.06 |
| Mean Effective Connectivity | From IPS to FEF | 1.00 | -0.19 |
| Mean Effective Connectivity | From FEF to THAL | 1.00 | -0.17 |
| Mean Effective Connectivity | From FEF to IPS | 1.00 | 0.13 |
| Mean Effective Connectivity | From FEF to FEF | 1.00 | -0.21 |
| Connection Type | Connection | BMA Probability | BMA parameter |
| Mean Modulation | From THAL to THAL | 1.00 | -1.47 |
| Mean Modulation | From THAL to IPS | 0.93 | 0.45 |
| Mean Modulation | From THAL to FEF | 1.00 | 0.99 |
| Mean Modulation | From IPS to THAL | 0.00 | -0.00 |
| Mean Modulation | From IPS to IPS | 1.00 | -0.47 |
| Mean Modulation | From IPS to FEF | 0.82 | 0.12 |
| Mean Modulation | From FEF to THAL | 0.00 | -0.00 |
| Mean Modulation | From FEF to IPS | 1.00 | 0.41 |
| Mean Modulation | From FEF to FEF | 1.00 | -0.54 |
| Connection Type | Connection | BMA Probability | BMA parameter |
| Group Differences in Mean Connectivity | From THAL to THAL | 0.71 | -0.06 |
| Group Differences in Mean Connectivity | From THAL to IPS | 0.73 | 0.05 |
| Group Differences in Mean Connectivity | From THAL to FEF | 1.00 | -0.17 |
| Group Differences in Mean Connectivity | From IPS to THAL | 0.00 | -0.00 |
| Group Differences in Mean Connectivity | From IPS to IPS | 1.00 | -0.10 |
| Group Differences in Mean Connectivity | From IPS to FEF | 1.00 | 0.08 |
| Group Differences in Mean Connectivity | From FEF to THAL | 0.00 | -0.00 |
| Group Differences in Mean Connectivity | From FEF to IPS | 0.00 | 0.00 |
| Group Differences in Mean Connectivity | From FEF to FEF | 0.73 | -0.05 |
| Connection Type | Connection | BMA Probability | BMA parameter |
| Group Differences in Modulation | From THAL to THAL | 0.00 | 0.00 |
| Group Differences in Modulation | From THAL to IPS | 1.00 | -0.59 |
| Group Differences in Modulation | From THAL to FEF | 0.00 | -0.00 |
| Group Differences in Modulation | From IPS to THAL | 0.00 | -0.00 |
| Group Differences in Modulation | From IPS to IPS | 0.00 | 0.00 |
| Group Differences in Modulation | From IPS to FEF | 0.00 | -0.00 |
| Group Differences in Modulation | From FEF to THAL | 0.00 | -0.00 |
| Group Differences in Modulation | From FEF to IPS | 0.00 | -0.00 |
| Group Differences in Modulation | From FEF to FEF | 0.74 | 0.26 |

| **Table S7.** SZ clinical analyses DCM parameters | | | |  |
| --- | --- | --- | --- | --- |
| SAPS Influence on Double-step modulation | | | | |
| Connection type | Connection | BMA Probability | BMA parameter | |
| Modulation due to Double-step | From THAL to THAL | 1.00 | -1.51 | |
| Modulation due to Double-step | From THAL to IPS | 0.00 | 0.00 | |
| Modulation due to Double-step | From THAL to FEF | 0.94 | 0.66 | |
| Modulation due to Double-step | From IPS to THAL | 0.00 | 0.00 | |
| Modulation due to Double-step | From IPS to IPS | 1.00 | -0.49 | |
| Modulation due to Double-step | From IPS to FEF | 0.92 | 0.18 | |
| Modulation due to Double-step | From FEF to THAL | 0.00 | 0.00 | |
| Modulation due to Double-step | From FEF to IPS | 1.00 | 0.60 | |
| Modulation due to Double-step | From FEF to FEF | 0.00 | 0.00 | |
| Connection type | Connection | BMA Probability | BMA parameter | |
| SAPS influence on Double-step Modulation | From THAL to THAL | 0.00 | 0.00 | |
| SAPS influence on Double-step Modulation | From THAL to IPS | 0.00 | 0.00 | |
| SAPS influence on Double-step Modulation | From THAL to FEF | 0.69 | 0.01 | |
| SAPS influence on Double-step Modulation | From IPS to THAL | 0.00 | 0.00 | |
| SAPS influence on Double-step Modulation | From IPS to IPS | 0.00 | 0.00 | |
| SAPS influence on Double-step Modulation | From IPS to FEF | 0.00 | 0.00 | |
| SAPS influence on Double-step Modulation | From FEF to THAL | 0.00 | 0.00 | |
| SAPS influence on Double-step Modulation | From FEF to IPS | 0.00 | 0.00 | |
| SAPS influence on Double-step Modulation | From FEF to FEF | 0.00 | 0.00 | |
| SANS Influence on Double-step modulation | | | | |
| Connection type | Connection | BMA Probability | BMA parameter | |
| Modulation due to Double-step | From THAL to THAL | 1.00 | -1.51 | |
| Modulation due to Double-step | From THAL to IPS | 0.81 | -0.57 | |
| Modulation due to Double-step | From THAL to FEF | 0.00 | 0.00 | |
| Modulation due to Double-step | From IPS to THAL | 0.00 | 0.00 | |
| Modulation due to Double-step | From IPS to IPS | 1.00 | -0.50 | |
| Modulation due to Double-step | From IPS to FEF | 0.00 | 0.00 | |
| Modulation due to Double-step | From FEF to THAL | 0.00 | 0.00 | |
| Modulation due to Double-step | From FEF to IPS | 1.00 | 0.60 | |
| Modulation due to Double-step | From FEF to FEF | 0.00 | 0.00 | |
| Connection type | Connection | BMA Probability | BMA parameter | |
| SANS influence on Double-step Modulation | From THAL to THAL | 0.00 | 0.00 | |
| SANS influence on Double-step Modulation | From THAL to IPS | 0.98 | 0.04 | |
| SANS influence on Double-step Modulation | From THAL to FEF | 1.00 | 0.04 | |
| SANS influence on Double-step Modulation | From IPS to THAL | 0.00 | 0.00 | |
| SANS influence on Double-step Modulation | From IPS to IPS | 0.00 | 0.00 | |
| SANS influence on Double-step Modulation | From IPS to FEF | 0.99 | 0.01 | |
| SANS influence on Double-step Modulation | From FEF to THAL | 0.00 | 0.00 | |
| SANS influence on Double-step Modulation | From FEF to IPS | 0.00 | 0.00 | |
| SANS influence on Double-step Modulation | From FEF to FEF | 0.00 | 0.00 | |
| SAPP Influence on Double-step modulation | | | | |
| Connection type | Connection | BMA Probability | BMA parameter | |
| Modulation due to Double-step | From THAL to THAL | 1.00 | -1.45 | |
| Modulation due to Double-step | From THAL to IPS | 0.00 | 0.00 | |
| Modulation due to Double-step | From THAL to FEF | 1.00 | 1.03 | |
| Modulation due to Double-step | From IPS to THAL | 0.00 | 0.00 | |
| Modulation due to Double-step | From IPS to IPS | 0.00 | 0.00 | |
| Modulation due to Double-step | From IPS to FEF | 0.00 | 0.00 | |
| Modulation due to Double-step | From FEF to THAL | 0.00 | 0.00 | |
| Modulation due to Double-step | From FEF to IPS | 1.00 | 1.07 | |
| Modulation due to Double-step | From FEF to FEF | 1.00 | -1.18 | |
| Connection type | Connection | BMA Probability | BMA parameter | |
| SAPP influence on Double-step Modulation | From THAL to THAL | 0.00 | 0.00 | |
| SAPP influence on Double-step Modulation | From THAL to IPS | 0.99 | 0.18 | |
| SAPP influence on Double-step Modulation | From THAL to FEF | 0.00 | 0.00 | |
| SAPP influence on Double-step Modulation | From IPS to THAL | 0.00 | 0.00 | |
| SAPP influence on Double-step Modulation | From IPS to IPS | 1.00 | -0.13 | |
| SAPP influence on Double-step Modulation | From IPS to FEF | 1.00 | 0.08 | |
| SAPP influence on Double-step Modulation | From FEF to THAL | 0.00 | 0.00 | |
| SAPP influence on Double-step Modulation | From FEF to IPS | 1.00 | -0.18 | |
| SAPP influence on Double-step Modulation | From FEF to FEF | 1.00 | 0.38 | |
| IPASE Influence on Double-step modulation | | | | |
| Connection type | Connection | BMA Probability | BMA parameter | |
| Modulation due to Double-step | From THAL to THAL | 0.00 | 0.00 | |
| Modulation due to Double-step | From THAL to IPS | 0.58 | -0.43 | |
| Modulation due to Double-step | From THAL to FEF | 0.00 | 0.00 | |
| Modulation due to Double-step | From IPS to THAL | 0.00 | 0.00 | |
| Modulation due to Double-step | From IPS to IPS | 0.74 | -0.54 | |
| Modulation due to Double-step | From IPS to FEF | 0.00 | 0.00 | |
| Modulation due to Double-step | From FEF to THAL | 0.00 | 0.00 | |
| Modulation due to Double-step | From FEF to IPS | 0.88 | 0.87 | |
| Modulation due to Double-step | From FEF to FEF | 0.56 | -0.37 | |
| Connection type | Connection | BMA Probability | BMA parameter | |
| IPASE influence on Double-step Modulation | From THAL to THAL | 0.00 | 0.00 | |
| IPASE influence on Double-step Modulation | From THAL to IPS | 0.00 | 0.00 | |
| IPASE influence on Double-step Modulation | From THAL to FEF | 0.00 | 0.00 | |
| IPASE influence on Double-step Modulation | From IPS to THAL | 0.00 | 0.00 | |
| IPASE influence on Double-step Modulation | From IPS to IPS | 0.00 | 0.00 | |
| IPASE influence on Double-step Modulation | From IPS to FEF | 0.00 | 0.00 | |
| IPASE influence on Double-step Modulation | From FEF to THAL | 0.00 | 0.00 | |
| IPASE influence on Double-step Modulation | From FEF to IPS | 0.00 | 0.00 | |
| IPASE influence on Double-step Modulation | From FEF to FEF | 0.00 | 0.00 | |
| CPZ Influence on Double-step modulation | | | | |
| Connection type | Connection | BMA Probability | BMA parameter | |
| Modulation due to Double-step | From THAL to THAL | 1.00 | -1.90 | |
| Modulation due to Double-step | From THAL to IPS | 0.00 | 0.00 | |
| Modulation due to Double-step | From THAL to FEF | 1.00 | 1.11 | |
| Modulation due to Double-step | From IPS to THAL | 0.00 | 0.00 | |
| Modulation due to Double-step | From IPS to IPS | 0.75 | -0.28 | |
| Modulation due to Double-step | From IPS to FEF | 0.00 | 0.00 | |
| Modulation due to Double-step | From FEF to THAL | 0.00 | 0.00 | |
| Modulation due to Double-step | From FEF to IPS | 0.99 | 0.61 | |
| Modulation due to Double-step | From FEF to FEF | 0.00 | 0.00 | |
| Connection type | Connection | BMA Probability | BMA parameter | |
| CPZ influence on Double-step Modulation | From THAL to THAL | 0.00 | 0.00 | |
| CPZ influence on Double-step Modulation | From THAL to IPS | 0.00 | 0.00 | |
| CPZ influence on Double-step Modulation | From THAL to FEF | 0.00 | 0.00 | |
| CPZ influence on Double-step Modulation | From IPS to THAL | 0.00 | 0.00 | |
| CPZ influence on Double-step Modulation | From IPS to IPS | 0.00 | 0.00 | |
| CPZ influence on Double-step Modulation | From IPS to FEF | 0.00 | 0.00 | |
| CPZ influence on Double-step Modulation | From FEF to THAL | 0.00 | 0.00 | |
| CPZ influence on Double-step Modulation | From FEF to IPS | 0.00 | 0.00 | |
| CPZ influence on Double-step Modulation | From FEF to FEF | 0.00 | 0.00 | |
| Note: The PEB and BMA model were only optimized over the B matrix, so A and C parameters are not estimated in the BMA. | | | | |

| **Table S8.** HC clinical analyses DCM parameters | | | |  |
| --- | --- | --- | --- | --- |
| IPASE Influence on Double-step modulation | | | | |
| Connection type | Connection | BMA Probability | BMA parameter | |
| Modulation due to Double-step | From THAL to THAL | 0.81 | -0.81 | |
| Modulation due to Double-step | From THAL to IPS | 0.95 | 1.25 | |
| Modulation due to Double-step | From THAL to FEF | 0.77 | 0.68 | |
| Modulation due to Double-step | From IPS to THAL | 0.00 | 0.00 | |
| Modulation due to Double-step | From IPS to IPS | 0.92 | -0.94 | |
| Modulation due to Double-step | From IPS to FEF | 0.00 | 0.00 | |
| Modulation due to Double-step | From FEF to THAL | 0.00 | 0.00 | |
| Modulation due to Double-step | From FEF to IPS | 0.00 | 0.00 | |
| Modulation due to Double-step | From FEF to FEF | 0.77 | -0.76 | |
| Connection type | Connection | BMA Probability | BMA parameter | |
| IPASE influence on Double-step Modulation | From THAL to THAL | 0.00 | 0.00 | |
| IPASE influence on Double-step Modulation | From THAL to IPS | 0.00 | 0.00 | |
| IPASE influence on Double-step Modulation | From THAL to FEF | 0.00 | 0.00 | |
| IPASE influence on Double-step Modulation | From IPS to THAL | 0.00 | 0.00 | |
| IPASE influence on Double-step Modulation | From IPS to IPS | 0.00 | 0.00 | |
| IPASE influence on Double-step Modulation | From IPS to FEF | 0.00 | 0.00 | |
| IPASE influence on Double-step Modulation | From FEF to THAL | 0.00 | 0.00 | |
| IPASE influence on Double-step Modulation | From FEF to IPS | 0.00 | 0.00 | |
| IPASE influence on Double-step Modulation | From FEF to FEF | 0.00 | 0.00 | |
| Note: The PEB and BMA model were only optimized over the B matrix, so A and C parameters are not estimated in the BMA. | | | | |

| **Table S9.** Region of interest percent signal change (double-step minus passive-viewing) correlations with symptom scores | | | | | |
| --- | --- | --- | --- | --- | --- |
|  | SANS | SAPS | SAPP | IPASE (SZ) | IPASE (HC) |
| Region | Rho (p-value) | Rho (p-value) | Rho (p-value) | Rho (p-value) | Rho (p-value) |
| FEF | 0.22 (0.251) | -0.02 (0.923) | 0.24 (0.228) | 0.05 (0.793) | 0.12 (0.568) |
| IPS | 0.27 (0.154) | -0.20 (0.297) | 0.07 (0.735) | -0.09 (0.670) | 0.02 (0.937) |
| THAL | -0.15 (0.430) | -0.10 (0.613) | 0.13 (0.502) | -0.04 (0.864) | 0.15 (0.493) |
| Notes: frontal eye field (FEF); intraparietal sulcus (IPS); thalamic nuclei associated with corollary discharge (THAL); the scale for assessment of positive symptoms total score (SAPS); the scale for assessment of negative symptoms total score (SANS); Inventory of Psychotic-like Anomalous Self-Experiences lifetime score (IPASE); the Scale for the Assessment of Passivity Phenomena lifetime score (SAPP) | | | | | |
